# Vitamin C, doxycycline, and azithromycin (VDA) targeted changes in cellular senescence-related genes in human adipose-derived mesenchymal stem cells

**DOI:** 10.22038/ijbms.2024.78183.16905

**Published:** 2024

**Authors:** Roshanak Alvandi, Samira Salimiyan, Mohammad Moradzad, Mobin Mohammadi, Shohreh Fakhari, Mohammad Reza Rahmani

**Affiliations:** 1 Student Research Committee, Kurdistan University of Medical Sciences, Sanandaj, Iran; 2 Department of Immunology, Faculty of Medicine, Kurdistan University of Medical Sciences, Sanandaj, Iran; 3 Department of Clinical Biochemistry, Faculty of Medicine, Kurdistan University of Medical Sciences, Sanandaj, Iran; 4 Cancer and Immunology Research Center, Research Institute for Health Development, Kurdistan University of Medical Sciences, Sanandaj, Iran; 5 Cellular and Molecular Research Center, Research Institute for Health Development, Kurdistan University of Medical Sciences, Sanandaj, Iran; 6 Zoonosis Research Center, Research Institute for Health Development, Kurdistan University of Medical Sciences, Sanandaj, Iran

**Keywords:** Adipose-derived - mesenchymal stem cells, Azithromycin, Cellular senescence, Combination drug therapy, Doxycycline, Regenerative medicine Vitamin C

## Abstract

**Objective(s):**

Adipose-derived Mesenchymal stem cells (ASCs) have garnered attention for their regenerative potential; therefore, their cellular senescence-related gene expression remains crucial in therapeutic contexts. Nowadays, combination therapies have shown promising results in reducing senescent cells. This study investigated the effects of vitamin C, doxycycline, and azithromycin co-treatment on the key cellular senescence-associated genes in ASCs.

**Materials and Methods:**

Human ASCs were cultured and treated for 24 hr with vitamin C, doxycycline, azithromycin, and a combination of three drugs. Total RNAs were extracted, and the expression of p21, p16, Nanog, Oct4, and Sox2 genes was assessed using reverse transcription-quantitative polymerase chain reaction (RT-qPCR). Additionally, cell cycle alterations were analyzed via flow cytometry after treatment with these compounds.

**Results:**

Notably, vitamin C treatment resulted in a significant down-regulation of p21 gene expression (*P*<0.01), implicating the potential role of vitamin C in promoting cell cycle progression. Doxycycline treatment led to a significant up-regulation of p21 and p16 gene expression (*P*<0.05), as it has previously been shown to induce cell cycle arrest. Similarly, azithromycin treatment predominantly increased p21 expression (*P*<0.05). Besides, cell cycle analysis revealed that each compound had changed the distribution of cells across different phases of the cell cycle.

**Conclusion:**

The combined use of all three drugs yielded intricate interactions, suggesting a complex yet promising approach to future research. According to our findings, the major difference in the combination drug-treated group (VDA) can be explained by the neutralizing effect of these three components in the environment.

## Introduction

Senescence is a process that cells apply in response to internal and external stimuli, reinforcing them to make irreversible blockade in the G1 phase in the cell cycle (1). The senescence process plays a crucial role in the accumulation of damaged and dysfunctional cells, contributing to tissue degeneration as well as impaired regenerative capacity, which is characterized as a potential risk factor for a range of diseases, such as stroke, Alzheimer’s, type 2 diabetes, and various cancers (2, 3). The central molecular mechanisms associated with the senescence process are increased β-galactosidase activity, reduced telomere length, and increased signaling pathways which are involved in regulating the cell cycle-related molecules namely, p53/p21 and p16/ Retinoblastoma protein (RBP) (4, 5). Although the senescence process occurs in differentiated cells, it can reduce stem cell differentiation, leading to a lower chance of being a better candidate for regenerative medicine purposes (6, 7). 

Amidst the cell types impacted by senescence, mesenchymal stem cells (MSCs) have been at the center of attention for regenerative medicine due to their remarkable properties, including self-renewal and multipotent differentiation potential (8). MSCs are found in various tissues, such as adipose tissue, bone marrow, amniotic fluid, umbilical cord, and synovial membrane, and actively contribute to tissue homeostasis, immune regulation, and tissue repair processes (9). Adipose-derived mesenchymal stem cells (ASCs) possess the unique ability to self-renew and differentiate into various mesodermal lineages, including adipocytes, osteoblasts, and chondrocytes (10). This remarkable plasticity and multilineage differentiation capacity render MSCs attractive candidates for regenerative medicine and cell-based therapy (11). Despite their regenerative potential, MSCs are subject to aging-related changes that negatively impact their functionality and regenerative abilities. The self-renewal and multilineage differentiation capacity decline as MSCs age, compromising their therapeutic efficacy (12). The senescence-related alterations in MSCs are associated with multiple changes in gene expression profiles and cellular signaling pathways (13).

Among the genes crucially involved in cellular senescence and pluripotency are p21 (14), p16 (15), SRY-box 2 (Sox2) (16), Octamer-binding transcription factor 4 (Oct4) (17), and Homeobox protein NANOG (Nanog) (18). Cell cycle progression, genomic integrity, and stem cell fate determination are all controlled by these key regulatory genes; However, their dysregulation has been implicated in the development of age-related diseases, tissue degeneration, and cellular senescence (19, 20). 

Pharmacological interventions have recently gained more attention as promising candidates for targeting cellular senescence (21). Vitamin C (L-ascorbic acid) is an essential nutrient for humans, acting as an anti-oxidant and cofactor for enzymes and genome regulation via the electron transport chain (22). It has been shown that in human bladder cancer EJ cells, a low dose of vitamin C reduces the cellular senescence phenotype in these cells by inhibiting the p38 kinase pathway downstream of the p53 molecule (23). Regulating the genome, vitamin C reduces the number of reactive oxygen species, regulates the genome, as well as reducing molecules involved in cellular senescence such as P53, HIF, and FOXO, increases self-renewal, and reduces cellular senescence-related processes in MSCs (24).

Doxycycline is a second generation of tetracycline antibiotics, which has antimicrobial effects along with other various processes (25, 26). Previous studies have shown that pre-treatment of doxycycline for 24 hr has anti-inflammatory effects by inhibiting NF-κB as well as anti-senescence effects by inhibiting SASP in cells such as human umbilical vein endothelial cells (HUVECs) (27, 28). 

Azithromycin, a macrolide antibiotic, inhibits the growth of a wide range of Gram-positive and Gram-negative bacteria (29, 30). Additionally, azithromycin has other functions that affect several processes along with anti-inflammatory features (31). Some studies considered azithromycin a senolytic drug through its ability to induce autophagy and senescent cell removal (32, 33). Recently, multiple studies indicated a promising outcome in prescribing a cocktail of pharmaceutical components to accelerate the remedy for cancer via eliminating cancer stem cells and senescent fibroblasts (33-35). For instance, a study investigated the simultaneous effect of vitamin C, doxycycline, and azithromycin treatment on cancer stem cells. They observed that azithromycin, which has anti-aging properties, combined with doxycycline and vitamin C in high concentrations leads to eradication of cancer stem cells (34).

In this study, we investigated whether exposure to vitamin C, doxycycline, and azithromycin (VDA) in combination differentially affected the anti-senescence properties of ASCs by measuring the changes in the cellular senescence-related genes. We aimed to explore the possible synergistic effects on cellular senescence in human adipose-derived MSCs. We saw that VDA can conversely modulate cellular senescence-related genes which shows the intricate interplay between these pharmacological agents on key regulatory genes involved in senescence and pluripotency. We speculate the neutralizing effects of these drugs combined have an important role in our findings. 

## Materials and Methods

***Isolation and culture of ASCs***

 ASCs were isolated from the omental adipose tissue of healthy individuals with their informed consent (Female 25–45 years old, IR.MUK.REC.1402.015). Adipose tissue samples were collected and transferred to the laboratory in Hanks basic salt solution buffer (HBSS) containing penicillin (300 U/ml), streptomycin (300 µg/ml), and amphotericin B (25 µg/ml). For non-enzymatic isolation of MSCs from adipose tissue, the tissue was minced into small pieces and placed in 6-well plates. Each well was supplemented with fetal bovine serum (FBS) (Life Technologies, United Kingdom) and incubated for 24 hr in a humidified incubator at 37 °C with 5% CO_2_. After 24 hr of incubation, the FBS in each well was removed, and low glucose Dulbecco’s modified Eagle’s media (DMEM-LG) (Life Technologies) containing penicillin (100 U/ml), streptomycin (100 µg/ml), and 10% FBS (complete regular culture media) was added. The media were changed every 48 hr. When the cells reached 80% confluence and exhibited a fibroblastic morphology, they were detached using 0.25% trypsin/EDTA and transferred to T25 flasks at a density of 10,000 cells/cm^2^. The cells were cultured until passage 5 for further experiments (36).

***Characterization of ASCs***

*Surface marker analysis*

To assess the surface markers of ASCs, human-monoclonal-conjugated antibodies with fluorescent tags were employed. The ASCs were detached using trypsin and resuspended in FACS Buffer (PBS+BSA 0.1%). The cells were then incubated in a dark place at 4 °C for 45 min with the following human-monoclonal-conjugated antibodies: CD45-FITC, CD105-FITC, CD73-PE, CD34-PE, and CD90-PerCP (BioLegend) (eBioscience). Mouse monoclonal antibodies conjugated with PE, FITC, and PerCP (eBioscience) were used as isotype controls. Following the incubation period, all groups were washed three times with FACS buffer and fixed in 4% paraformaldehyde. Fluorescence intensity was measured using a flow cytometer (FACS Calibur; Becton Dickinson), and the data was analyzed with FlowJo V.7.6 software (FlowJo LLC, Ashland, OR, USA) (37).

*Differentiation assays (adipogenic, osteogenic, and chondrogenic differentiation)*

To investigate the multipotent differentiation potential of ASCs, we performed adipogenic, osteogenic, and chondrogenic induction assays using specific culture media and differentiation protocols, as we have previously reported (37).

*Adipogenic differentiation*

For adipogenesis, the regular media was replaced with the StemPro adipogenic differentiation media (Life Technologies). The cells were incubated in the adipogenic media for 21 days with regular media change intervals every 72 hr. Successful adipogenic differentiation was confirmed after staining the cells with oil red O solution and visualization of lipid droplets using a light microscope (Olympus, Japan).

*Osteogenic differentiation*

To induce osteogenesis, the regular media was replaced with the StemPro osteogenic differentiation media (Life Technologies). The cells were cultured in the osteogenic media for 21 days, with media renewal every 72 hr. Successful osteogenic differentiation was ultimately validated by alizarin red S staining, which detects calcium deposits in the extracellular matrix of cells.

*Chondrogenic differentiation*

To promote chondrogenesis, ASCs were cultured with StemPro chondrogenic differentiation media (Life Technologies) for 14 days with regular media change intervals every 72 hr. Successful chondrogenic differentiation was determined by alcian blue staining, which detects proteoglycan-rich extracellular matrix produced by cells.

***Cell*** ***viability assa*y**

To provide general insights into the impact of different concentrations of vitamin C, doxycycline, and azithromycin on the survival of ASCs, we carried out ASCs viability assay. First, we performed a (4,5dimethylthiazol-2-yl)-2,5-diphenyltetrazolium bromide (MTT) assay on ASCs treated with different concentrations of vitamin C, doxycycline, and azithromycin ASCs were seeded at a density of 5×10³ cells per well in a 96-well plate. The following day, cells were treated with different concentrations of vitamin C (12.5, 25, 50, 100, and 200 µg/ml), doxycycline (0.1, 0.05, 0.025, 0.0125, and 0.00625 µg/ml), and azithromycin (0.25, 0.125, 0.062, 0.031, and 0.015 µg/ml) in DMEM+ FBS (10%) for 24 hr. After the incubation period, the drug-containing media was aspirated, and 120 µl of MTT solution (5 mg/ml) was added to each well, followed by incubation for 2 to 4 hr in a 5% CO₂ incubator at 37 °C with 95% humidity. Subsequently, the MTT-containing media was removed, and 100 µl of dimethyl sulfoxide (DMSO) was added to dissolve the formazan crystals. The plate was further incubated for 15 min, and the absorbance of the resulting solution was measured at 550 nm using a microplate reader (Synergy HTX). Cell viability was calculated as a percentage relative to the untreated control cells (38).

***Study design and cell treatment***

To assess the effect of vitamin C, doxycycline, azithromycin, and a combination of these three drugs (VDA) treatment on cellular senescence-related genes and cell cycle of ASCs in all experimental groups, cells from passage 5 were cultured separately for 24 hr with 25 µg/ml vitamin C, 0.1 µg/ml doxycycline, 0.125 µg/ml azithromycin, and VDA in complete media. To provide a basis for comparison, untreated ASCs were used as the control group. After 24 hr, cells were harvested for assessment of gene expression and flow cytometry.

***Beta-galactosidase assay***

To evaluate beta-galactosidase activity using cell event senescence green flow cytometry assay kit (Invitrogen, US), a cellular suspension from all groups (treated and untreated) containing 10^6^ cells/ml is meticulously prepared in PBS. Subsequently, 100 µl of this cell suspension was carefully transferred to flow tubes and centrifuged at 1500 RPM, 5 min, at 14 °C. Once the supernatant was discarded, for permeabilizing the cells, 100 µl of 2% paraformaldehyde solution was promptly added, initiating a 10-minute dark incubation at room temperature. Following the incubation, the cells were washed using 1% BSA solution, ensuring the removal of any extraneous particles. A 100 µl aliquot of the working solution is meticulously added to each sample, which is then incubated at 37 °C without CO_2_, for 2 hr. After the incubation, the cells undergo another complete wash with 1% BSA to eliminate any residual traces of the incubation media. The cells are then gently resuspended in a 1% BSA solution. This detailed procedure culminates with the flow cytometric analysis of the samples within the FL1 channel, yielding valuable insights into beta-galactosidase activity as an outcome of the experimental intervention. We assessed Beta-Galactosidase activity using a flow cytometer (FACS Calibur; Becton Dickinson), and eventually the data were analyzed with FlowJo V.7.6 software (FlowJo LLC, Ashland, OR, USA).

***Gene expression analysis by RT-qPCR***

*RNA extraction and cDNA synthesis*

For gene expression analysis, total RNA was extracted from the cultured ASCs using an RNA extraction kit (SINACLON, Iran) according to the manufacturer’s instructions. The extracted RNA was quantified using a spectrophotometer (Synergy HTX), and its purity was assessed by measuring the absorbance ratio at 260/280 nm. Complementary DNA (cDNA) was synthesized using 1 µg of total RNA via a high-capacity cDNA synthesis kit (SINACLON, Iran). The reverse transcription reaction was performed in a thermal cycler with the following conditions for 40 cycles: 25 °C for 10 min, 50 °C for 50 min, and 70 °C for 15 min. The resulting cDNA was stored at -20 °C until further use.

*RT-qPCR*

Real-time quantitative PCR (RT-qPCR) was performed using specific primers for the target genes: β-actin, Octamer-binding transcription factor 4 (Oct4), SRY-box 2 (Sox2), Homeobox protein NANOG (Nanog), p21, and p16. All primers were designed using Gene Runner software version 5.0.78.0 and their specificity was confirmed by the National Center for Biotechnology Information database (NCBI) (Table 1). The expression of these genes in all experimental groups was analyzed through real-time PCR. The prepared samples from all experimental groups in the SYBR green master mix (SINACLON, Iran) were evaluated using the Rotor-Gene^TM^ 6000 sequence detection system (Corbett Life Science, Australia). The thermal cycling conditions included an initial denaturation step at 95 °C for 10 min, followed by 40 cycles of denaturation at 95 °C for 15 sec, and annealing/extension at the specified temperatures for 30 sec. 

Subsequently, the expression levels of target genes were determined and normalized to a reference gene (β-actin) using standard curves and the 2^-ΔΔCt^ formula (39). ΔC_t_ was calculated as follows: 

∆∆CT= ΔCT target samples [CT target gene - CTβ-actin] - ΔCT reference samples [CT target gene - CTβ-actin]

For the comparison of gene expression levels among different groups, a one-way analysis of variance (ANOVA) was performed, followed by *post hoc* multiple comparisons, using Tukey’s test to identify significant differences between each group.

***Cell cycle analysis***

To investigate the cell cycle, we utilized propidium iodide (PI) staining. Briefly, a cell suspension of 1 × 10^6^ cells/ml was prepared and washed with 0.1% BSA. Following this, the cells were fixed with 70% cold ethanol and incubated at 4 °C for 1 hr. After fixation, the cells were carefully washed with 0.1% BSA. Then the cell pellet was resuspended in 1 ml of sodium citrate solution containing 50 µg/ml PI and 50 µl of 100 µg/ml RNase A and incubated at 4 °C overnight. Analysis was performed using a flow cytometer (FACS Calibur; Becton Dickinson) and the data were analyzed with FCS Express 7 research edition. Mean values of cell cycle phases among different groups were compared via ANOVA followed by Tukey’s *post hoc* test.

***Statistical analysis***

The statistical analysis for this study was carried out using the software package GraphPad Prism 3.02. Data were analyzed using SPSS V.16 software (IBM Analytics). The results were considered statistically significant at a *P*-value of less than 0.05; *P*<0.05 and *P*<0.01 are depicted in Figures by * and**. 

## Results

***Characteristics of ASCs***

**Figure 1:**
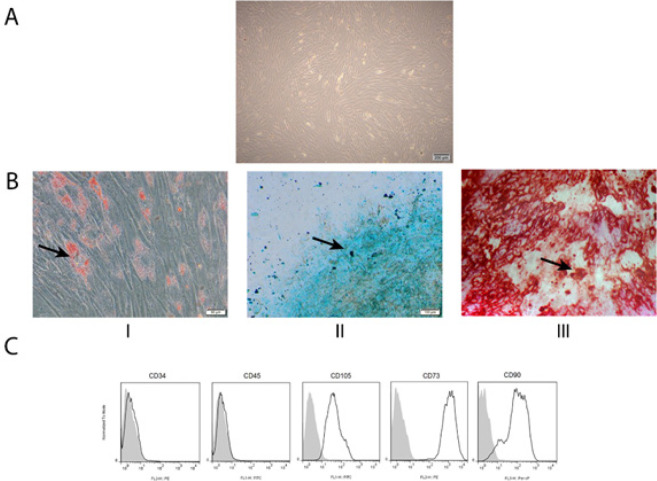
Adipose-derived mesenchymal stem cell (ASCs) characteristics

The isolated cells from adipose tissue samples were spindle-shaped and showed plastic adherent characteristics (Figure 1A). In addition, these cells were able to differentiate into osteogenic, adipogenic, and chondrogenic lineages (Figure 1B). Besides, flow cytometry results showed that isolated cells were positive for CD90, CD73, and CD105, and negative for CD45 and CD34 markers (Figure 1C). 

***Beta-galactosidase enzyme activity assessment***

**Figure 2:**
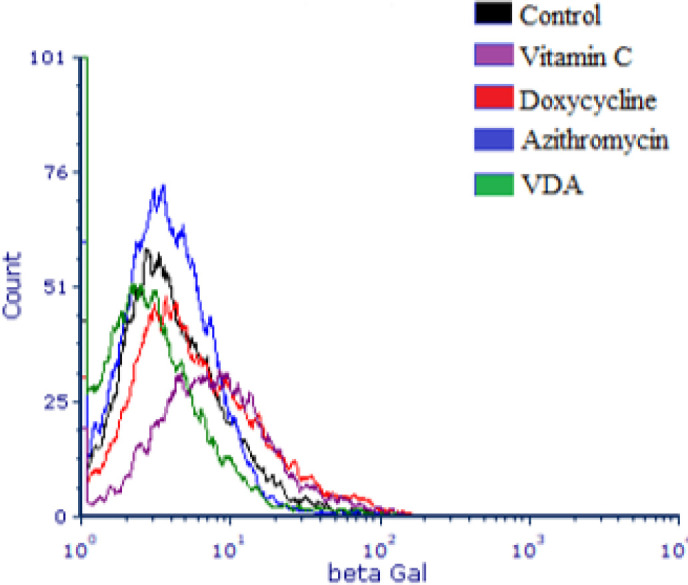
Beta-galactosidase enzyme activity assessment

In general, we observed that the results of beta-galactosidase showed no significant difference in this study (*P*>0.05). Flow cytometric analysis was performed, and Figure 2 illustrates the beta-galactosidase enzyme activity in different treatment groups compared to the control group.

***RT-qPCR analysis for p16 and p21 genes***

**Figure 3:**
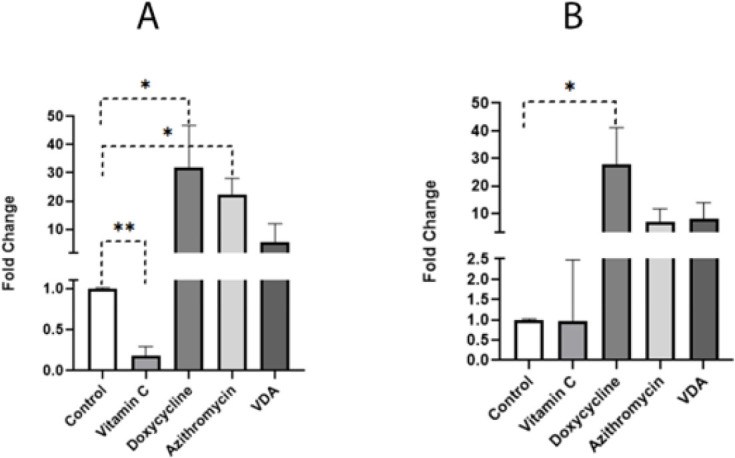
A) Relative gene expression of p21 Gene. (B) Relative gene expression of p16 Gene. The expression ratio is illustrated as the mean ± SD of the 2^-ΔΔCt^ formula. b-Actin was utilized as a reference gene in this calculation. *Indicates a significance level of below 0.05.

The analysis of gene expression using RT-qPCR showed changes among the treated groups. The mean expression level of the *p21* gene in vitamin C, doxycycline, azithromycin, and VDA in the groups were 0.19±0.09, 32.01±12, 22.43±4.53, and 5.56±1.27, respectively. The expression ratio of this gene was lower in the vitamin C group compared to the control group (*P*<0.01). *p21* expression in the doxycycline and azithromycin groups was higher than in the control group (*P*<0.05). Although *p21* expression in the VDA group was altered in comparison with the control group, it wasn’t statically significant (*P*>0.05) (Figure 3A). The mean expression levels of the *p16* gene in vitamin C, doxycycline, azithromycin, and VDA groups were 0.96±1.23, 27.88±10.83, 7.02±3.80, and 8.17±4.74, respectively. The expression ratio of this gene was significantly higher in the doxycycline-treated group compared to the control group (*P*<0.05). *p16* expression in other treated groups was not significantly changed in comparison with the control group (*P*>0.05) (Figure 3B). The mean expression levels of the *SOX2* gene in vitamin C, doxycycline, azithromycin, and VDA groups were 1.03±0.74, 2.40±1.22, 0.99±0.69, and 2.13±0.75, respectively. The expression ratio of this gene was insignificantly changed in all treated groups compared to the control group (*P*>0.05). The mean expression levels of the *OCT4* gene in vitamin C, doxycycline, azithromycin, and VDA groups were 1.57±0.97, 0.64±0.07, 0.95±0.29, and 0.79±0.44, respectively. The expression ratio of this gene showed no significant changes (*P*>0.05). The mean expression levels of the *NANOG* gene in vitamin C, doxycycline, azithromycin, and VDA groups were 0.99±0.30, 2.26±0.06, 1.13±0.35, and 2.26±2.14, respectively. The expression ratio of this gene remained almost unchanged in vitamin C, doxycycline, azithromycin, and VDA groups compared to the control group (*P*>0.05).

***Cell cycle analysis***

**Figure 4:**
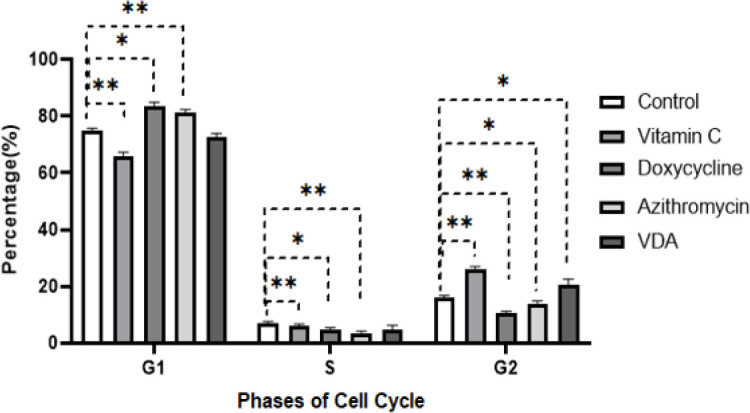
Mean percentages of cells in different cell cycle phases

**Figure 5:**
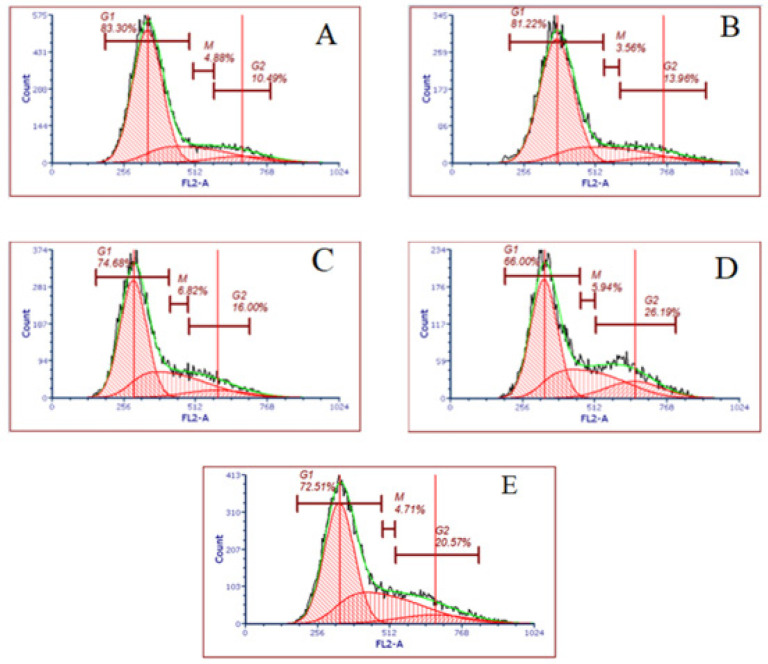
Cell cycle flow cytometry results

The impact of the studied interventions on cell cycle progression was assessed using flow cytometry. The mean percentages of cells in different cell cycle phases are summarized in Figure 4, revealing distinct alterations in the distribution of cell cycle phases among the groups. The mean percentage of cells in the G1 phase in control, vitamin C, doxycycline, azithromycin, and VDA in the groups were 74.68% ± 0.90, 66.01% ± 0.97, 83.31% ± 1.28, 81.22% ±0.90, and 72.52% ± 1.15, respectively. The mean percentage of cells in the G1 phase in the vitamin C group was lower than the control group (*P*<0.01). Conversely, the doxycycline group displayed a noticeable increase in cell population in the G1 phase compared with the control group (*P*<0.05). Similarly, the azithromycin group exhibited an elevated G1 phase population compared to the control group (*P*<0.01). Intriguingly, the VDA group did not exhibit any substantial alterations in cell cycle distribution compared to the control group (*P*>0.05). The graphical representation of these findings is depicted in Figure 5, where distinct shifts in cell cycle phases among the studied groups are visually evident.

## Discussion

The investigation of compounds capable of modulating cellular senescence-related gene expression in MSCs holds significant promise for regenerative medicine and therapeutic interventions. In this study, we explored the effects of well-known antibiotics, including doxycycline and azithromycin, along with vitamin C, on the expression of key genes involved in the senescence process in ASCs.

**Figure 6:**
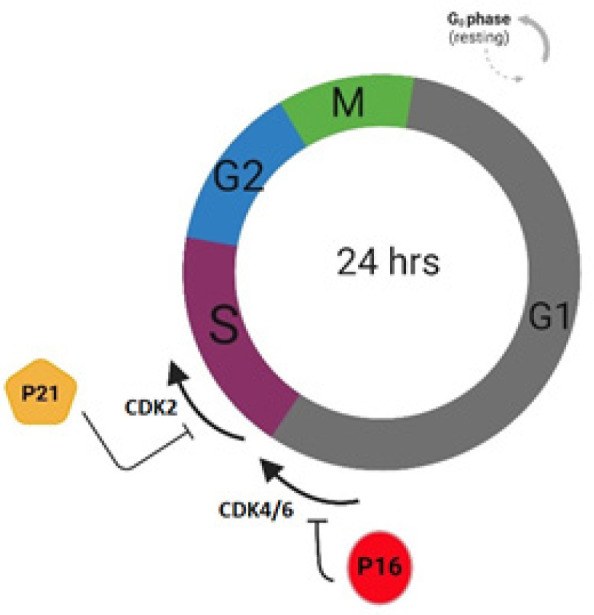
A schematic overview of the cell cycle phases and the roles of p16 and p21 in cell cycle arrest

Our findings reveal that doxycycline treatment itself led to a substantial up-regulation of p21 and p16 gene expression in ASCs in comparison with the untreated ones. This observation aligns with existing literature that highlights doxycycline’s capacity to induce cell cycle arrest in pancreatic cancer cells (PANC-1) (40). This becomes particularly relevant in light of the role of p21 and p16 in inhibiting CDK2 and CDK4/6, respectively (4), resulting in G1 phase cell cycle arrest and prevention of the G1/S transition (Figure 6). The concurrent flow cytometry analysis substantiates these findings, demonstrating an accumulation of cells in the G1 phase upon doxycycline treatment. Although published evidence reported anti-senescence properties for doxycycline by inhibiting factors involved in cellular senescence (27, 28), as well as the increasing self-renewal ability of pluripotent stem cells (41), our results indicate a potential paradox by suggesting doxycycline’s capability to induce cell cycle arrest in ASCs. The reason for these various effects was not elucidated within the result of this study due to the complexity of function and its involvement in various signaling pathways in the cytosol of cells (25). NF-κB signaling pathway, a critical regulator of cellular structure, exhibits an escalating activity with aging (42). Doxycycline, in turn, modulates this pathway, altering its activity (28). Thus, this signaling cascade could explain the diverse effects of the drug on cells.

Similarly, the up-regulation of p21 in the azithromycin group highlights a potential mechanism by which azithromycin may impact cell cycle progression, which warrants further investigation. The observed distinction in p16 expression between the doxycycline and azithromycin groups is noteworthy, suggesting that while both compounds impact the cellular senescence-related gene expression, their specific mechanisms may differ. In our study flow cytometry analysis showed a significant contribution of azithromycin in increasing the population of arrested cells in the G1 phase. While Qiu *et al.* revealed that azithromycin reduces senescence phenotype by autophagy induction in senescent mRC-5 lung fibroblast cells (32), we observed that it can push ahead the senescence process by increasing p21 gene expression. In parallel to the aforementioned doxycycline’s role in various signaling pathways in the cytosol, azithromycin can also exert such an effect in human bronchial epithelial cells (43). Studies have indicated that azithromycin inhibits NF-κB signaling (44). Therefore, this impact may be responsible for the various effects of azithromycin. 

Conversely, our results revealed a significant down-regulation of p21 gene expression in the vitamin C-treated group, confirming its role in promoting cell cycle progression, and impact on cell cycle regulation (45). Additionally, cell cycle analysis delineated a decrease in the cell population treated with vitamin C in the G1 Phase, indicating anti-senescence properties of vitamin C in ASCs. This intriguing observation indicates a plausible anti-senescence effect of vitamin C in the ASCs, emphasizing its capacity to influence the balance between cellular quiescence and division. These findings align with previous research by Kim *et al.* where it was demonstrated that vitamin C contributes to cell cycle progression by enhancing p21/p53 signaling pathways (23). Our study extends this understanding, presenting compelling evidence of vitamin C’s potential as a modulator of senescence-related processes in ASCs.

The impact of the VDA yielded intriguing results. Although each compound (vitamin C, doxycycline, and azithromycin) had discernible effects on gene expression and the cell cycle individually, their combined impact in the VDA group seemed to be less significant. The lack of significant changes in the expression of the examined genes in the VDA group, as well as the absence of noticeable shifts in cell cycle distribution, might suggest intricate interactions among these compounds within the context of ASCs. The complexity of these interactions may result in a neutralizing effect, dampening the individual impact of each drug. This insight contributes to a comprehensive understanding of the subtle interplay between compounds within complex biological systems, shedding light on potential challenges and opportunities for therapeutic applications in future studies, which yet need to be further investigated.

 Intriguingly, our investigation did not reveal appreciable differences in β-galactosidase activity among the various treatment groups. While this outcome might initially seem contrary to expectations (45), it is essential to consider the condition and exposure time in our study. The relatively short-term treatment window employed in our experimental design might have conferred insufficient time for notable changes in β-galactosidase activity to emerge. Cellular senescence is a multifaceted process intricately linked with cumulative cellular stresses (1), and its manifestation could require more protracted exposure time windows to these treatments under investigation.

Furthermore, the RT-qPCR analysis targeting the expression levels of Nanog, Oct4, and Sox2 in the treated groups yielded interesting results. These crucial pluripotency-associated genes are renowned for their pivotal roles in maintaining stemness and cellular identity (20). Our findings, however, indicated a lack of noticeable differences in the expression patterns of these genes across the various treatment conditions. This observation opens avenues for insightful speculation. The PI3K/ Akt pathway is a signaling pathway that has an important role in the self-renewal and pluripotency of ASCs and affects the expression of Nanog, Oct4, and Sox2 expression (41). It is plausible that no significant changes in the expression of these 3 genes are due to this pathway that we did not investigate. Furthermore, the relatively concise 24-hour experimental timeframe may have constrained our ability to capture significant fluctuations in the expression levels of Nanog, Oct4, and Sox2. 

 Overall, in this study, we found that the major difference in the combination drug-treated group (VDA) may have been related to the possible neutralizing effect of the interaction between these three components. On one hand, doxycycline and azithromycin increased the expression of p21 and p16, which made the ASCs stay in the G1 phase. On the other hand, vitamin C decreased the gene expression for p21 which resulted in removing the arrest of ASCs in G1 and ultimately proceeding to the S phase. Both notions were also confirmed in our flow cytometry results. Understanding how these genes work together, especially when influenced by subtle changes, might need various time exposure to uncover meaningful differences. As part of our future experiments, we will continue to examine the different time exposures to these drugs as well as the possible cellular pathways involved in causing the ASCs to show this phenotype.

**Table 1:** Primer sequences and their respective product sizes along with annealing temperatures of β-actin, OCT4, SOX2, NANOG, P21, P16

*Genes*	*Forward primer*	*Reverse primer*	*Product size (bp)*	*Annealing Tm*
*β-actin*	*GCCTTTGCCGATCCGC*	*GCCGGAGCCGTTGTCG*	*90*	*63.1*
***OCT4***	***TTGGGCTCGAGAAGGATGTG***	***TGAGAAAGGAGACCCAGCAG***	***119***	***63.1***
*SOX2*	*ATGGGTTCGGTGGTCAAGTC*	*GCTCTGGTAGTGCTGGGACA*	*183*	*63.1*
*NANOG*	*GAATCTTCACCTATGCCTGTG*	*GTTGTTTGCCTTTGGGACTG*	*169*	*57.5*
*p21*	*CAGCATGACAGATTTCTACC*	*CACACAAACTGAGACTAAGG*	*147*	*56.5*
*p16*	*AAGGTCCCTCAGACATCC*	*ATGGACATTTACGGTAGTGG*	*216*	*57.5*

β-actin: beta-actin, OCT4: octamer-binding transcription factor 3'4, SOX2: SRY-box 2, NANOG: homeobox protein NANOG

(A) The representative inverted microscope image of ASCs in passage 5 demonstrated their spindle-shaped morphology and plastic adherence. (B) Three-lineage differentiation of ASCs, presence of fat vacuoles, proteoglycans, and calcium deposits, respectively (shown by black arrows) were characterized by specific staining after culturing ASCs in differentiation media, confirming their mesenchymal stem cell nature. (C) Flow cytometry analyses confirmed isolated ASCs were positive for CD90, CD73, and CD105 and negative for CD45 and CD34.

CD: cluster of differentiation

The graph displays the results of the beta-galactosidase enzyme activity assessment in different experimental groups compared to the control group. Flow cytometry data show no significant differences in enzyme activity between the groups, indicating a consistent enzyme activity level across all conditions.

VDA: vitamin C, doxycycline, azithromycin

VDA: vitamin C, doxycycline, azithromycin

The percentage of cells that are arrested in the G1, S, and G2 stages of the cell cycle has been depicted. The percentage of cells arrested in all three stages significantly changed in single drug-treated groups, while the VDA-treated group remained intact in G1 and S, only significantly higher in G2. **indicates a significance level of below 0.01, and *indicates a significance level of below 0.05.

VDA: vitamin C, doxycycline, azithromycin

(A) The doxycycline group, (B) The azithromycin group, (C) The control group, (D) The vitamin C group, and (E) The VDA group. As it can be seen explicitly, the A and B groups show cell arrest in the G1 phase while the D group shows a reduced cell population in the G1 phase, and there is no significant change in the cell cycle induced in the E group.

VDA: vitamin C, doxycycline, azithromycin

CDK: cycline-dependent kinase

## Conclusion

The results of this study shed light on the complex interplay between these compounds and cellular processes. Doxycycline and azithromycin exhibited distinct impacts on p21 and p16 expression, hinting at potential roles in senescence modulation. In contrast, vitamin C demonstrated anti-senescence potential through p21 down-regulation and cell cycle modulation. The interaction of the 3-drug combination adds a layer of complexity, revealing intriguing dynamics of “neutralizing effects”. However, the intricate nature of pluripotency gene expression and the short-term nature of our study emphasize the need for comprehensive investigations and longer-duration studies. These insights offer new directions for regenerative medicine and therapeutic interventions. Evaluating the biological effects of these three drugs on senescence is complex, given their ability to impact a wide array of biological pathways. Nevertheless, studying the effects of VDA on senolysis represents a starting point for further elucidating drug mechanisms in senescence, offering potential avenues for valuable insights for our future experiments.
